# Epidemiology of mild cognitive impairment in Brazil

**DOI:** 10.1590/S1980-57642013DN74000002

**Published:** 2013

**Authors:** Sonia Maria Dozzi Brucki

**Affiliations:** 1 MD, PhD. Cognitive Neurology and Behavioural Group. FMUSP. São Paulo SP, Brazil.

**Keywords:** mild cognitive impairment, epidemiology, prevalence, incidence, Brazil

## Abstract

With the worldwide increase in longevity and rising prevalence of cognitive disorders in the aged population, efforts have been made to characterize mild cognitive impairment (MCI) and its prevalence and/or incidence in a number of countries, given MCI may be a pre-dementia phase of degenerative conditions. The aim of this review was to retrieve the available data on the prevalence and incidence of mild cognitive impairment (MCI) in Brazil and compare these with rates found by studies conducted in other countries. The Pubmed and Scielo databases were searched using the following search terms: mild cognitive impairment, prevalence, incidence, including studies in both English and Portuguese languages. Only one study on MCI prevalence has been published in Brazil, reporting a prevalence rate of 6.1% and incidence of 13.2/1000 persons-year among those aged 60 years or over. Prevalence rates for other countries are also reported. The prevalence and incidence of MCI found in Brazil is similar to rates observed in other countries.

## Abstract (pt)

Com o aumento da longevidade mundial e o crescimento da prevalência de distúrbios cognitivos nesta população, tem-se tentado caracterizar o comprometimento cognitivo leve e sua prevalência e/ou incidência nos diversos países. Uma vez que, este quadro pode ser uma fase pré-demencial dos quadros degenerativos. Esta revisão tentou recolher os dados disponíveis quanto a prevalência e incidência de comprometimento cognitivo leve (CCL) no Brasil e comparar em relação aos demais estudos em outros países. Foram revistas as bases PubMed e Scielo com os termos: comprometimento cognitivo leve, prevalência, incidência, foram incluídos estudos em inglês, português. Apenas um estudo de prevalência de CCL foi publicado no Brasil, com taxa de 6,1% e incidência de 13,2/1000 pessoas-ano entre aqueles de 60 anos ou mais. São mostradas as taxas nos demais países. A prevalência e incidência de CCL no Brasil encontram-se dentro das faixas observadas entre outros países.

## INTRODUCTION

The aging of the world population has been extensive and constantly disseminated by surveys and media channels. The importance of this phenomenon centers largely on the burden that it places on the world. Concomitant with aging, besides retirement from work and the costs of pension payments, are the disease most commonly associated with aging, including dementia, amongst others. Many countries have attempted to improve control of age-related chronic non-communicable diseases, which are associated with increased morbimortality.

According to data from the World Health Organization (WHO), between 2000 and 2050, the proportion of the world population over 60 years of age is set to double from 11% to 22% (605 million to over two billion). An estimated 80% of elderly in the world will reside in low and medium-income countries, placing a heavy burden on the governments of these countries. Life expectancy in Brazil has risen from 67 years in 1990 to 73 years in 2009. The population aged 65 years or older has grown in recent decades; at the last census in 2010, this group represented 7.4% of the population but exhibited a heterogeneous distribution by region (ranging from 4.6 in the Northern region to 8.1% in the South and Southeast).

More recently, attention has been focused on milder cognitive disorders. The concept of mild cognitive impairment has been used in population-based studies in recent years.

The concept of Mild Cognitive Impairment (MCI) emerged to classify individuals who are at an intermediate stage between normal cognition and dementia, more specifically AD.^1^

Initially, the diagnostic criteria of MCI emphasized memory impairment and defined a risk factor for progression to AD. MCI was later recognized as a heterogeneous entity in terms of its clinical presentation, etiology and prognosis, whereby deficits in other cognitive domains, besides memory, became accepted with the resultant division of MCI into subtypes. Thus, four subtypes have been defined: amnestic MCI single domain, amnestic MCI multiple domains, nonamnestic MCI single domain, and nonamnestic MCI multiple domains.^2^

This objective of this review was to retrieve the available data on the prevalence and incidence of MCI in Brazil and compare these with rates found by studies conducted in other countries.

## METHODS

Literature searches were performed on PubMed and Scielo databases using the search terms "mild cognitive impairment", "epidemiology", "prevalence", and "incidence" for studies published between 1994 and September 2013. We searched the reference lists of retrieved papers to identify additional articles. Only full-text papers written in English, Portuguese, Spanish, and French were considered.

**Prevalence.** The estimated prevalence of MCI among elderly over 70 years of age is 14 to 18%.^3^ This prevalence can vary depending on the criteria adopted, types of tests employed and their cut-off values, as well as the age and educational level of the population under study. In a recent meta-analysis, prevalence ranged from 3% to 42%.^4^

In community-based studies, the prevalence of amnestic MCI ranged from 2.1% to 11.5%.^5^ A Korean study using Peterson's criteria found a prevalence of 28.6%,^6^ whereas a Mexican study using the same criteria but different instruments reported a prevalence of 6.45%.^7^ An intermediate rate was observed in a study conducted in Germany, with a prevalence of 7.8% using the original criteria and 12.1% when cognitive impairment complaints were excluded.^8^ In a meta-analysis of Chinese studies, MCI prevalence in the elderly population was 12.7% with a higher rate among illiterates.^9^ The prevalence in Italy was 6.0%, with the most frequent type being nonamnestic MCI single domain.^10^ In a rural area of Japan, MCI prevalence was high (23.5%), while the most frequent type was nonamnestic.^11^

In the population-based 10/66 study, the prevalence of amnestic MCI was between 0.8% in China and 4.3% in India (the study included low and middle-income countries). A total of 15376 participants were assessed, where scores 1.5 standard deviations below age- and schooling-adjusted means were considered abnormal memory performance.^5^

The prevalence of MCI in Medellin (Colombia) was 9.7%.^12^

The analysis of the literature available yielded only one study on MCI in Brazil, published by Godinho et al.^13^ based on data from the city of Porto Alegre (in the Southern region of Brazil). The study found a prevalence of MCI of 6.1% (n=21), 24% remained stable while 38% improved during follow-up treatment; the annual rate of conversion from MCI to AD was 8.5%.

Some authors of epidemiological studies have adopted the cognitive impairment no dementia (CIND) criteria, in which the individual has cognitive impairment yet without loss of functioning, not requiring confirmation by a companion or long-term follow-up.

In Brazil, surveys have been grounded in the concept of cognitive and functional impairment (which encompasses cognitive impairment, dementia and functional loss of all causes).^14^

Preliminary data from the study by César et al. (2013) revealed a CIND prevalence in the city of Tremembé (hinterland of São Paulo State – Southeastern region) of 18.6%.^15^

In the population-based PIETA study in elderly aged 75 or over, the prevalence of CIND was 25.2% and associated factors were advanced age, low socioeconomic level, depression and history of thyroid dysfunction.^16^

In a riverside-dwelling population with low schooling and practically no vascular risk factors, CIND prevalence was 7.7%. Unlike the other studies cited, this investigation was conducted in a rural zone among individuals aged 50 or older.^17^

In the United States, the prevalence of CIND in those over 70 years of age was 22.2%, ranging from 16% (71 to 79 years) to 39% (≥90 years).^18^

In the United States and Canada, prevalence ranged from 17 to 23% whereas in Europe the rate ranged from 21 to 27%. In published reviews, rates ranged from 5 to 29%.^19-26^ The average prevalence among Afro-Americans was 23.4% (rising with age, from 19.2% to 38%).^26^

In the city of Guadalajara in Mexico, the prevalence was 13.8% in a sample whose educational level varied widely from zero to 23 years of schooling and averaging 4.4 years, where 23% had no formal education. The factors associated with cognitive impairment were low schooling, age over 75 years, being unmarried, and depression. The greatest risk factor was educational level (OR=9.06); with a prevalence of 21% among those with zero to four years of schooling.^27^

In Taiwan, a prevalence of 9.7% was found among 6192 elderly subjects (aged over 65 years), with schooling exerting a major influence.^28^

In an Italian study conducted in Florence, CIND incidence was 21.37/1000 persons-year^18^ (DiCarlo et al., 2007).

**Incidence.** Incidence studies are generally harder to perform since they require greater time and resources (both financial and human) to conduct. Consequently, fewer incidence studies are available compared to prevalence studies, which represent a snapshot of a particular moment in time. Prevalence is the result of incidence (the development of new cases of a given disease) and survival time with the disease. If a disease has less evolution and greater mortality, it will have a lower prevalence in the population. Incidence studies are better for assessing the actual situation of the population regarding the disease.

The annual incidence of MCI is around 5% in individuals over 65 years of age.^29^ The incidence of amnestic MCI ranges from 9.9 to 40.6/1000 persons-year and of nonamnestic forms from 28 to 36.3/1000 persons-year. According to the systematic review by Luck et al. (2010), the incidence for all forms of MCI ranged from 51 to 76.8/1000 persons-year, where the main risk factors for conversion were age, low schooling, and arterial hypertension.^30^ In a more recent systematic review, an incidence of 21.5-71.3/1000 persons-year was identified for MCI and 8.5-25.9/1000 persons-year for amnestic MCI.^4^

In a population-based cohort study following around 2000 individuals for up to four years, approximately 30% of the participants developed MCI.^31^

The only study in Brazil including MCI incidence data showed a rate of 13.2/1000 persons-year among those aged 60 years or over, after an 8-year follow-up. Risk factors detected were educational level and initial scores on the Min-Mental State Exam (MMSE), with age not proving significant.^32^

## DISCUSSION

In general, MCI and CIND are more frequent than dementia, since the majority of individuals are at a pre-clinical stage with greater risk of conversion to dementia, whereas others never convert to dementia constituting a larger number of individuals in this state. The number is higher owing to the fact that this condition can persist for many years, with no clearly defined ratio for conversion to dementia. This impairment may be attributable to other non-degenerative causes such as those which are vasculatures, secondary to systemic diseases, metabolic and deficiency-induced; or even secondary to psychiatric conditions such as depression. Therefore, its frequency may be greater and fluctuate over time. The concept seems more suitable for use in epidemiological studies because it does not require confirmation of cognitive decline over time or confirmation by the companion, as suggested for diagnosing mild cognitive impairment. Therefore, it represents a more accurate concept for cross-sectional studies with less likelihood of bias.

We noted that the prevalence of MCI in our milieu lies midway between the rates of other countries for the over 60 age group. In studies assessing older populations, rates were found to be higher. Taking CIND into consideration gave a rate of between 7.7% and 25.2%. The studies retrieved differed in terms of risk factors involved, research setting, and chiefly in the age of subjects assessed. The prevalence in Brazil is similar to rates found for other populations.

Reanalyzing the incidence data, only the study involving a sample from the city of Porto Alegre provides this information, whose incidence was found to be lower than rates reported in a recent systemic review (Ward).

In conclusion, further studies of the Brazilian population are needed, controlling differently for risk factors, diet, educational levels, genetic background, among others. Studies of prevalence and incidence should be carried out in a continuous and repeated manner throughout the year, since today's risk factors may not be the same as those of tomorrow.
